# Interruption of schistosomiasis transmission in mountainous and hilly regions with an integrated strategy: a longitudinal case study in Sichuan, China

**DOI:** 10.1186/s40249-017-0290-6

**Published:** 2017-04-07

**Authors:** Yang Liu, Bo Zhong, Zi-Song Wu, Song Liang, Dong-Chuan Qiu, Xiao Ma

**Affiliations:** 1 Department of Health Education, West China School of Public Health, Sichuan University, No. 16 Renmin South Road, Chengdu, 610041 Sichuan Province China; 2 Sichuan Provincial Center for Disease Control and Prevention, No. 6 Zhongxue Road, Chengdu, 610041 Sichuan Province China; 3 Department of Environmental & Global Health, College of Public Health and Health Professions, University of Florida, Gainesville, FL USA

**Keywords:** Schistosomiasis japonica, Transmission interruption, Integrated strategy, Longitudinal effectiveness, Source of infection, Sichuan Province, Mountainous and hilly region

## Abstract

**Background:**

Schistosomiasis remains a major public health concern in China. Since 2004, an integrated strategy was developed to control the transmission of *Schistosoma japonicum* in China. However, the long-term effectiveness of this integrated strategy for the interruption of schistosomiasis transmission remains unknown in the mountainous and hilly regions of China until now. This longitudinal study aims to evaluate the effectiveness of the integrated strategy on transmission interruption of schistosomiasis in Sichuan Province from 2005 through 2014.

**Methods:**

The data regarding replacement of bovines with machines, improved sanitation, access to clean water, construction of public toilets and household latrines, snail control, chemotherapy, and health education were captured from the annual report of the schistosomiasis control programmes in Sichuan Province from 2005 to 2014, and *S. japonicum* infection in humans, bovines and snails were estimated to evaluate the effectiveness of the integrated strategy.

**Results:**

During the 10-year period from 2005 through 2014, a total of 536 568 machines were used to replace bovines, and 3 284 333 household lavatories and 15 523 public latrines were built. Tap water was supplied to 19 116 344 residents living in the endemic villages. A total of 230 098 hm^2^ snail habitats were given molluscicide treatment, and 357 233 hm^2^ snail habitats received environmental improvements. There were 7 268 138 humans and 840 845 bovines given praziquantel chemotherapy. During the 10-year study period, information, education and communication (IEC) materials were provided to village officers, teachers and schoolchildren. The 10-year implementation of the integrated strategy resulted in a great reduction in *S. japonicum* infection in humans, bovines and snails. Since 2007, no acute infection was detected, and no schistosomiasis cases or infected bovines were identified since 2012. In addition, the snail habitats reduced by 62.39% in 2014 as compared to that in 2005, and no *S. japonicum* infection was identified in snails since 2007. By 2014, 88.9% of the endemic counties achieved the transmission interruption of schistosomiasis and transmission control of schistosmiasis was achieved in the whole province in 2008.

**Conclusion:**

The government-directed and multi-department integrated strategy is effective for interrupting the transmission of schistosomiasis in the mountainous and hilly regions of China.

## Background

Schistosomiasis ranks second only to malaria among the tropical parasitic diseases of its significant economic and public health consequences [1]. Worldwide, it is estimated that more than 200 million people are living with this infectious disease of poverty, with over 800 million at risk of infection [2]. In China, schistosomiasis remains a major public health concern nowadays [3].

Based on geographic, ecologic, and epidemiologic profiles, the schistosomiasis endemic foci are classified into three types in China, including marshland and lake regions, plain regions with waterway networks, and mountainous and hilly regions [4]. Unlike in marshland and lake regions and plain regions with waterway networks, the intermediate host *Oncomelania hupensis* snails are mainly distributed in ditches and rice paddy in mountainous and hilly regions of China [5]. Such a feature complicates the snail control efforts, either by using molluscicide treatment or environmental improvement [6].

From the initiation of the schistosomiasis control activities in 1950s until now, the schistosomiasis control strategy has shifted four times, with adaptation to local socio-economic and epidemiologic factors, including control of infected humans and livestock in 1950s, snail elimination-based integrated strategy from 1960s to early 1980s, chemotherapy-based integrated strategy from late 1980s to 2003, and the currently implemented integrated strategy with emphasis on controlling the sources of *Schistosoma japonicum* infection since 2004 [7].

Sichuan Province is located in southwestern China, and it is the most afflicted mountainous region by *S. japonicum* in the country, which has the largest snail habitats and most severe morbidity due to *S. japonicum* [8]. Historically, schistosomiasis was endemic in 63 counties of the province, with more than 10 million people at risk of infection [8]. The control efforts since 1950s, notably the implementation of the chemotherapy-based integrated strategy since 1980s, had greatly reduced the prevalence and intensity of *S. japonicum* infection in Sichuan Province; however, the termination of the World Bank Loan Project for Chinese Schistosomiasis Control Program (WBLP), reform of specialized schistosomiasis institutions and reduced financial support to schistosomiasis control [9, 10], resulted in a re-emergence of schistosomiasis in the province at early 2000s [11]. Since 2004, a new government-directed and multi-department integrated strategy was therefore proposed for elimination of schistosomiasis [12]. Here, we present the results from a 10-year longitudinal study pertaining to the effectiveness of this integrated strategy on elimination of schistosomiasis in Sichuan Province from 2005 through 2014.

## Methods

### Data collection

The data regarding replacement of bovines with machines, improved sanitation, access to clean water, construction of public toilets and household latrines, snail control, chemotherapy, and health education were captured from the annual report of the schistosomiasis control programmes in Sichuan Province from 2005 to 2014 [13–22].

### Snail survey

At spring and autumn from 2005 to 2014, a snail survey was performed in historical snail habitats by means of the systematic sampling [13]. All snails captured in the field were transferred to laboratory, and identified for survival or death, and *S. japonicum* infection under a microscope [23]. The area of snail habitats, area with infected snails and snail infection rate were estimated.

### Detection of *S. japonicum* infection in humans and bovines

During the non-transmission period from 2005 to 2014, all residents living in the villages endemic for *S. japonicum* were detected for specific IgG antibodies against *S. japonicum* with a diagnostic kit for *Schistosoma* antibody (ScAb) by colloidal gold method (Sichuan Maccura Biotechnology Co., Ltd.; Chengdu, China) [24–26]. Then, all sero-seropositive subjects were detected for *S. japonicum* infections with a miracidium hatching testing [27]. In addition, all bovines in the villages endemic for *S. japonicum* were detected for *S. japonicum* infection with a miracidium hatching test [28].

### Ethical statement

This study was approved by the Ethical Review Committee of Sichuan Provincial Center for Disease Control and Prevention. All studies were performed in accordance with the international and national guidelines.

### Data management

All data were processed with the software Microsoft Excel version 2007 (Microsoft Corporation; Redmond, WA, USA).

## Results

### Implementation of integrated control interventions

During the 10-year period from 2005 through 2014, a total of 536 568 machines were used to replace bovines, and 3 284 333 household lavatories and 15 523 public latrines were built, including three-cell septic tanks and marsh-gas pools. In addition, we supplied tap water to 19 116 344 residents living in the endemic villages (Table 1). A total of 230 098 hm^2^ snail habitats were given molluscicide treatment, and 357 233 hm^2^ snail habitats received environmental improvement. There were 7 268 138 humans and 840 845 bovines given praziquantel chemotherapy. During the 10-year study period, information, education and communication (IEC) materials were provided to village officers, teachers and schoolchildren (Table 2).

**Table 1:** Improvement of sanitation and water resources in endemic areas from 2005 to 2014

Year	Non-hazardous toilets					Safe water resources			
	No. home lavatories	No. pubilc latrines	No. cumulative household with non-hazardous toilets	No. household in endemic areas	Coverage rate (%)	No. people with safe water	No. cumulative people with safe water	No. people in endemic areas	Coverage rate (%)
2005	64 056	52	293 065	505 284	58.00	1 177 177	5 374 517	9 696 043	55.43
2006	161 359	8 453	353 964	589 940	60.00	1 494 462	5 764 536	9 973 246	57.80
2007	216 304	56	437 850	695 000	63.00	1 446 709	6 477 187	10 363 500	62.50
2008	287 599	1 484	589 201	906 463	65.00	1 681 425	6 959 626	10 434 222	66.70
2009	260 626	212	658 611	997 895	66.00	2 071 942	7 242 199	10 465 606	69.20
2010	647 712	938	815 726	1 199 597	68.00	2 151 305	7 619 722	10 582 948	72.00
2011	444 231	1 120	915 067	1 307 238	70.00	2 006 939	8 380 644	10 675 980	78.50
2012	486 441	1 395	1 039 799	1 444 165	72.00	2 586 650	9 200 619	10 837 008	84.90
2013	461 253	1 709	1 028 186	1 370 914	75.00	2 201 601	9 912 597	11 001 773	90.10
2014	254 752	104	936 054	1 215 654	77.00	2 298 134	10 909 059	11 246 453	97.00

**Table 2:** Effectiveness of Health Education in Mountainous and hilly regions of Sichuan, China from 2005 to 2014

Year	Health education materials	Medium publicity	Training for village officers	Training for teachers	Protection products	Posters	Slogan	Prevention and control knowledges			
								Awareness rate (%)		Correct behavior formation rate (%)	
								Children	Women	Children	Women
2005	1 743 865	2 483	466	2 688	11 063	6 334	4 151				
2006	1 746 208	2 520	583	2 614	9 087	2 939	4 206				
2007	1 668 622	2 559	506	2 685	5 407	2 433	10 175				
2008	2 355 221	2 755	490	2 784	6 077	2 800	7 973	91.5	90.50	83.1	80.5
2009	2 591 166	2 576	518	2 836	7 018	2 283	9 872				
2010	1 759 749	2 786	507	2 723	18 036	2 700	10 179				
2011	3 660 317	2 589	5 544	2 793	19 455	2 617	8 837				
2012	4 074 518	2 649	6 545	2 752	21 158	2 706	6 890				
2013	4 627 189	2 629	6 562	2 726	24 618	4 597	8 283				
2014	4 417 960	2 614	5 524	2 765	22 948	4 089	7 910	96.85	95.01	96.31	94.09

### *S. japonicum* infection in human and bovines in Sichuan Province from 2005 to 2014

**Fig. 1:**
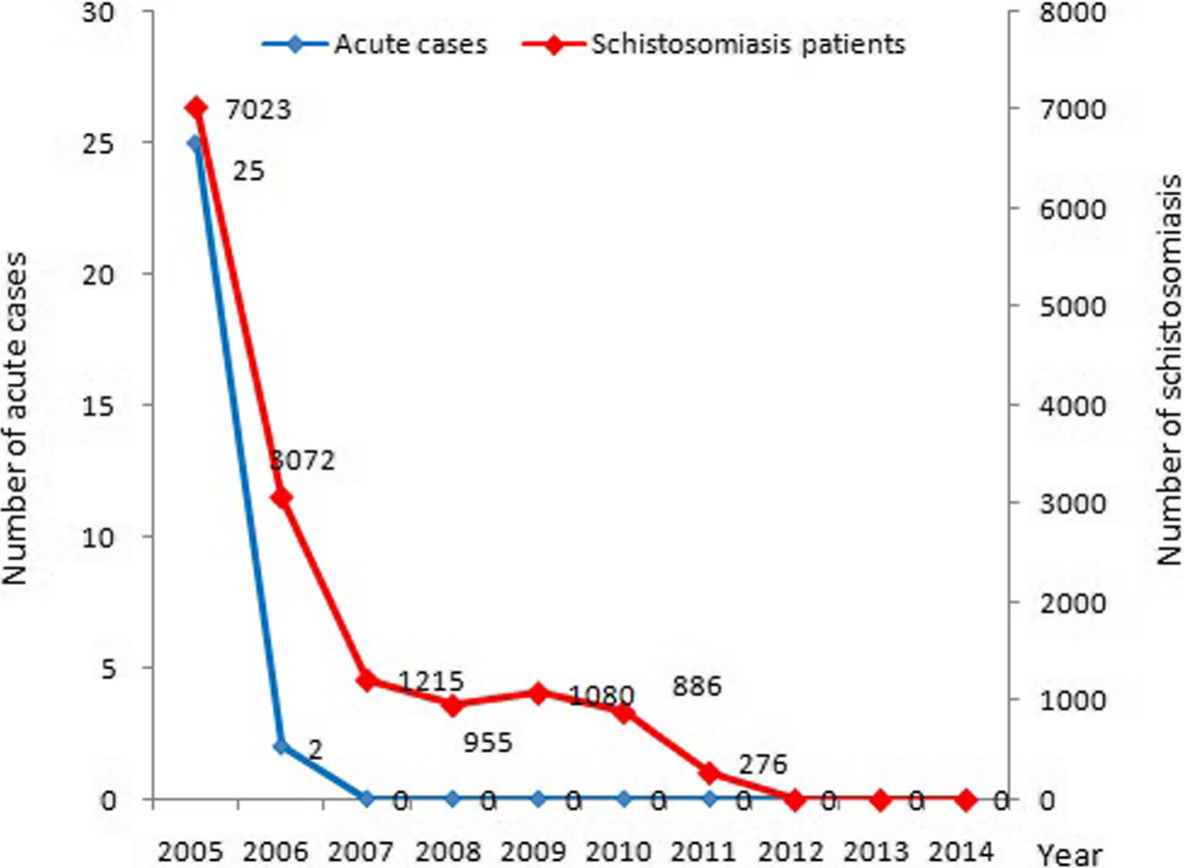
Number of schistosomiasis cases and acute cases in Sichuan Province from 2005 to 2014

During the 10-year longitudinal study period from 2005 to 2014, a total of 22 539 043 residents participated in the serological test, and 1 780 163 sero-positive individuals were subject to the miracidium hatching test. There were 7 023, 3 072, 1 215, 955, 1 080, 886, and 276 cases with schistosomiasis identified from 2005 to 2011, respectively; and no cases were detected since 2012 (Fig. 1). There was a tendency towards a decline in both the prevalence of *S. japonicum* human infection and the positive rate of serological test. There were 34 and 2 acute cases reported in 2005 and 2006, and no acute infections were detected since 2007.

**Fig. 2:**
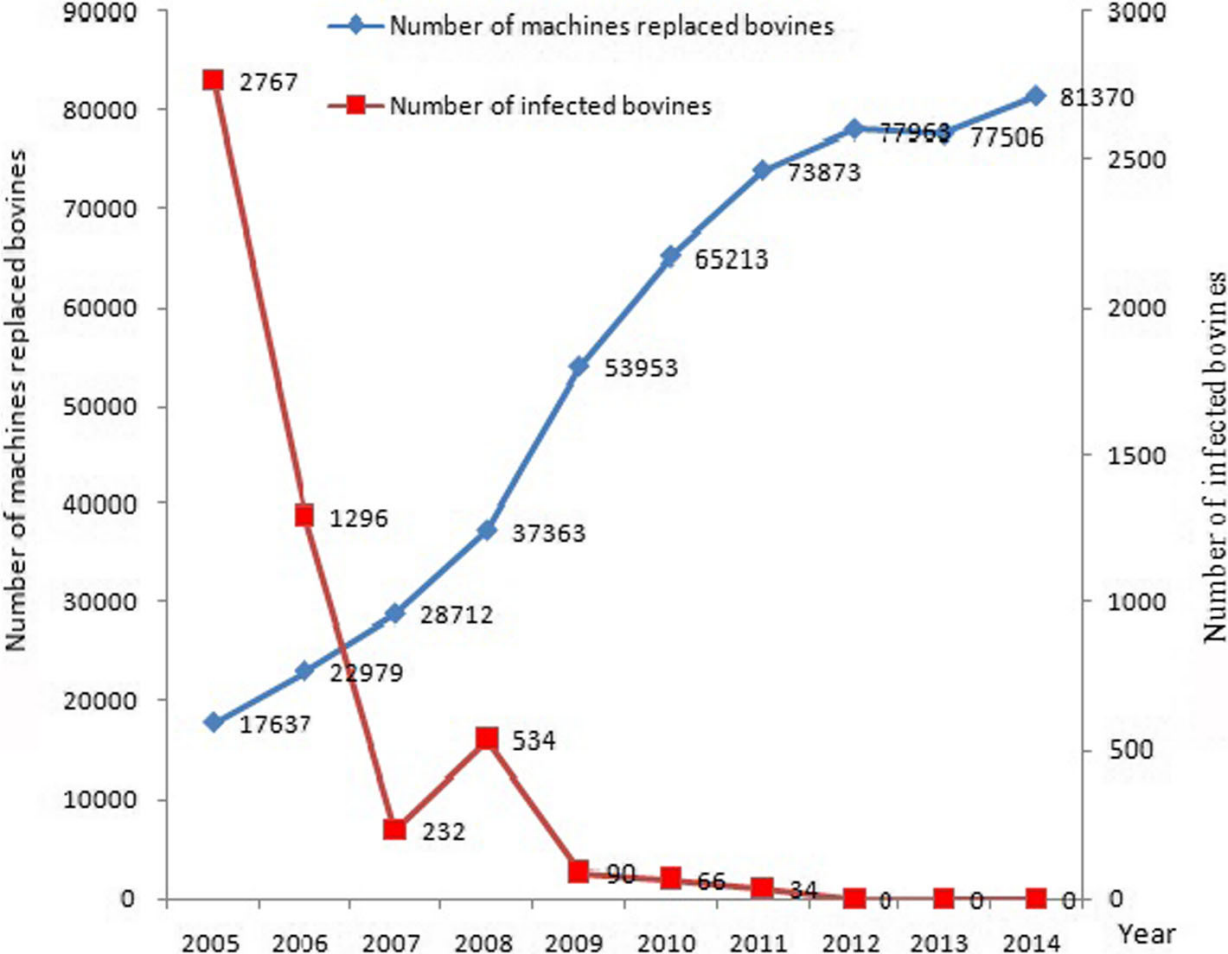
Number of bovines replaced by machines and *S. japonicum*-infected bovines in Sichuan Province from 2005 to 2014

From 2005 to 2011, a total of 2 767, 1 296, 232, 534, 90, 66, and 34 bovines were identified with *S. japonicum* infections, and no infection was found in bovines since 2012 (Fig. 2). A tendency towards a decrease was seen in the rate of *S. japonicum* infection in bovines across the study period.

### Dynamic shifts of snail status in Sichuan Province from 2005 to 2014

**Fig. 3:**
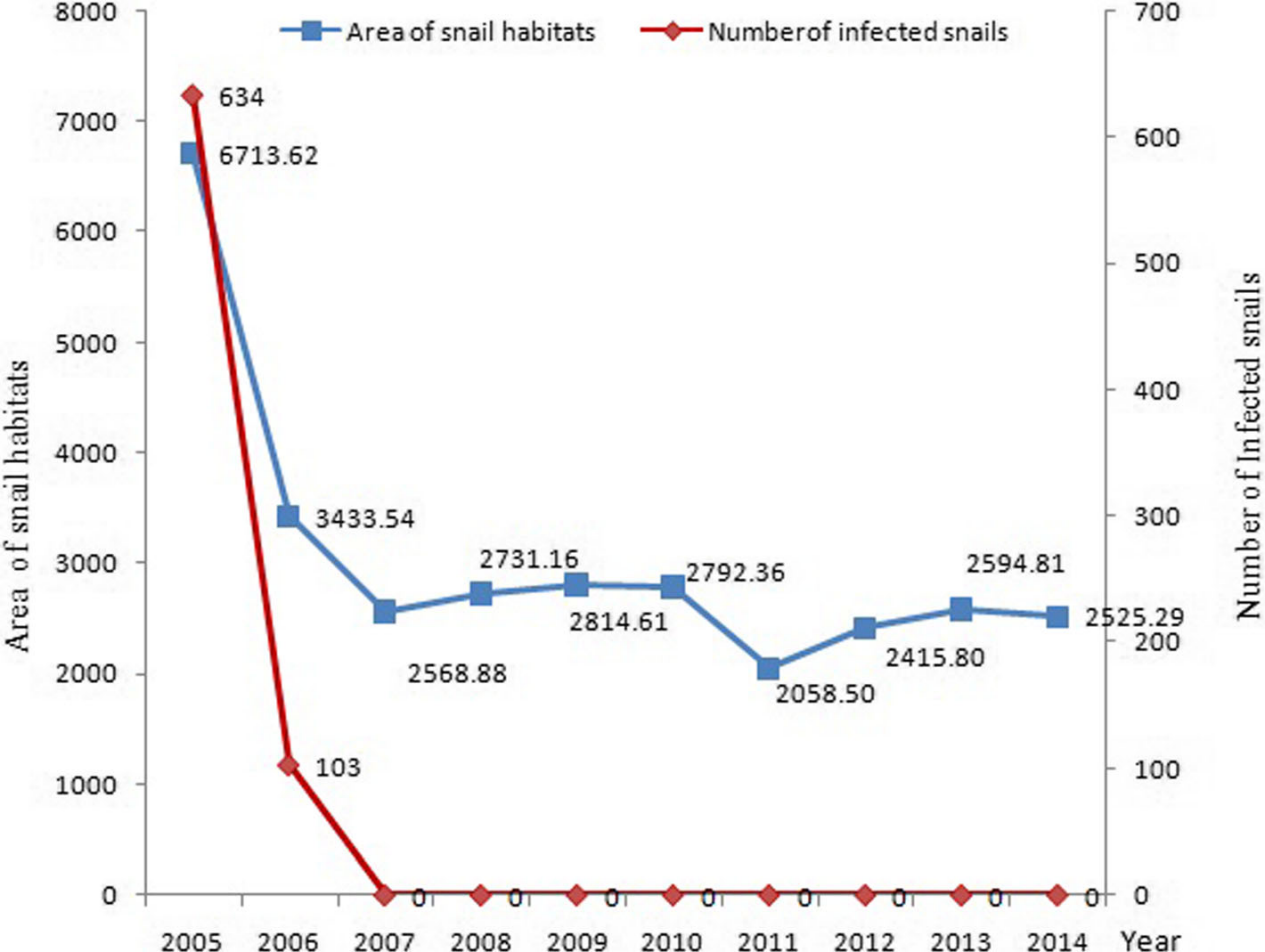
Annual area of snail habitats and annual number of *S. japonicum*-infected snails in Sichuan Province from 2005 to 2014

During the 10-year longitudinal study period from 2005 to 2014, snail surveys were conducted at an area of 390 157 hm^2^, and 247 494 hm^2^ area was subject to snail control with molluscicide treatment and environmental improvement. During the 10-year period, the snail habitats fluctuated from 2 058.5 to 6 713.62 hm^2^, and the snail habitats reduced by 62.39% in 2014 as compared to that in 2005, and no *S. japonicum* infection was identified in snails since 2007 (Fig. 3).

### Overall status of schistosomiasis control in Sichuan Province from 2005 to 2014

**Fig. 4:**
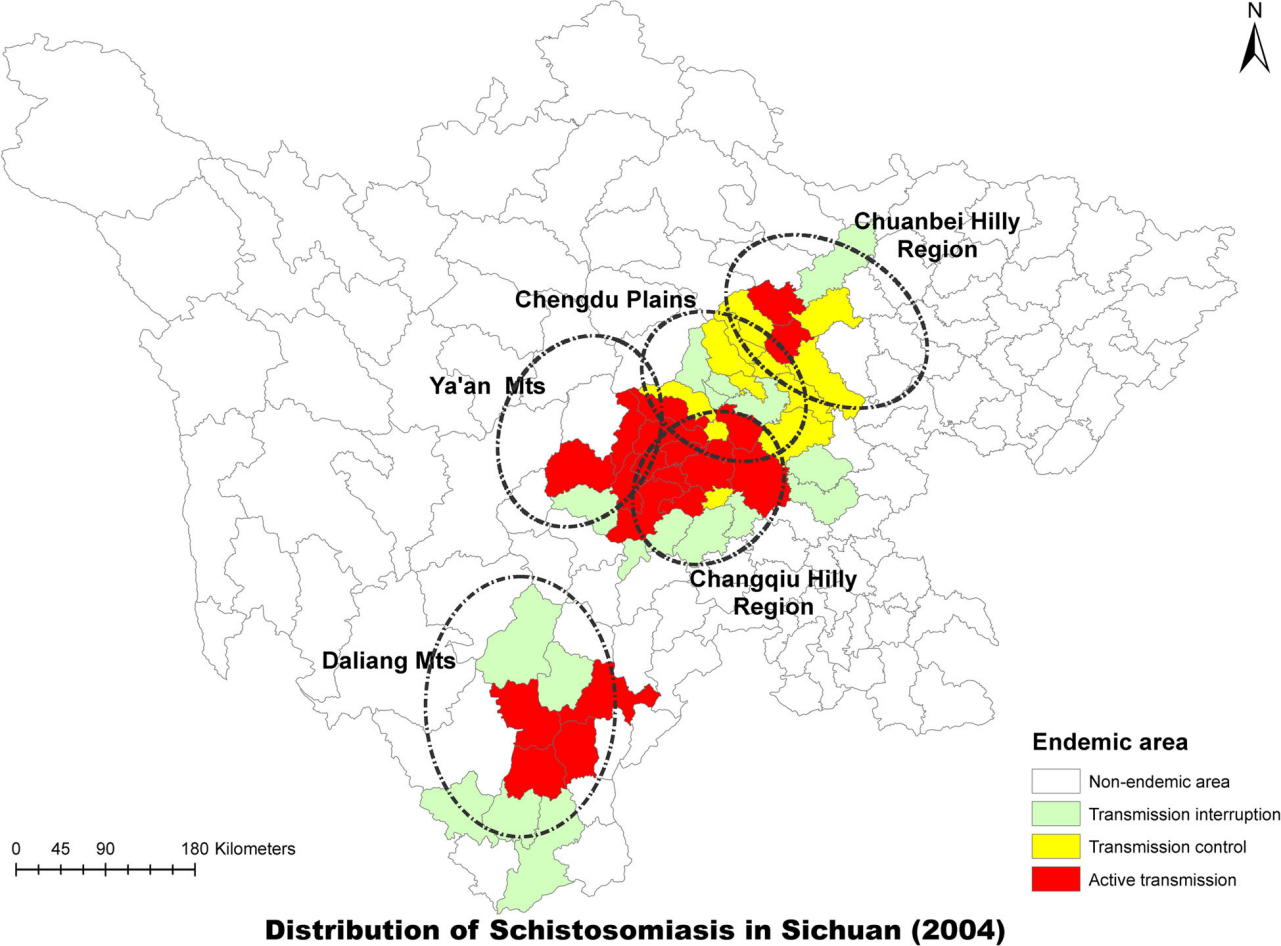
Distribution of schistosomiasis in Sichuan Province in 2004

**Fig. 5:**
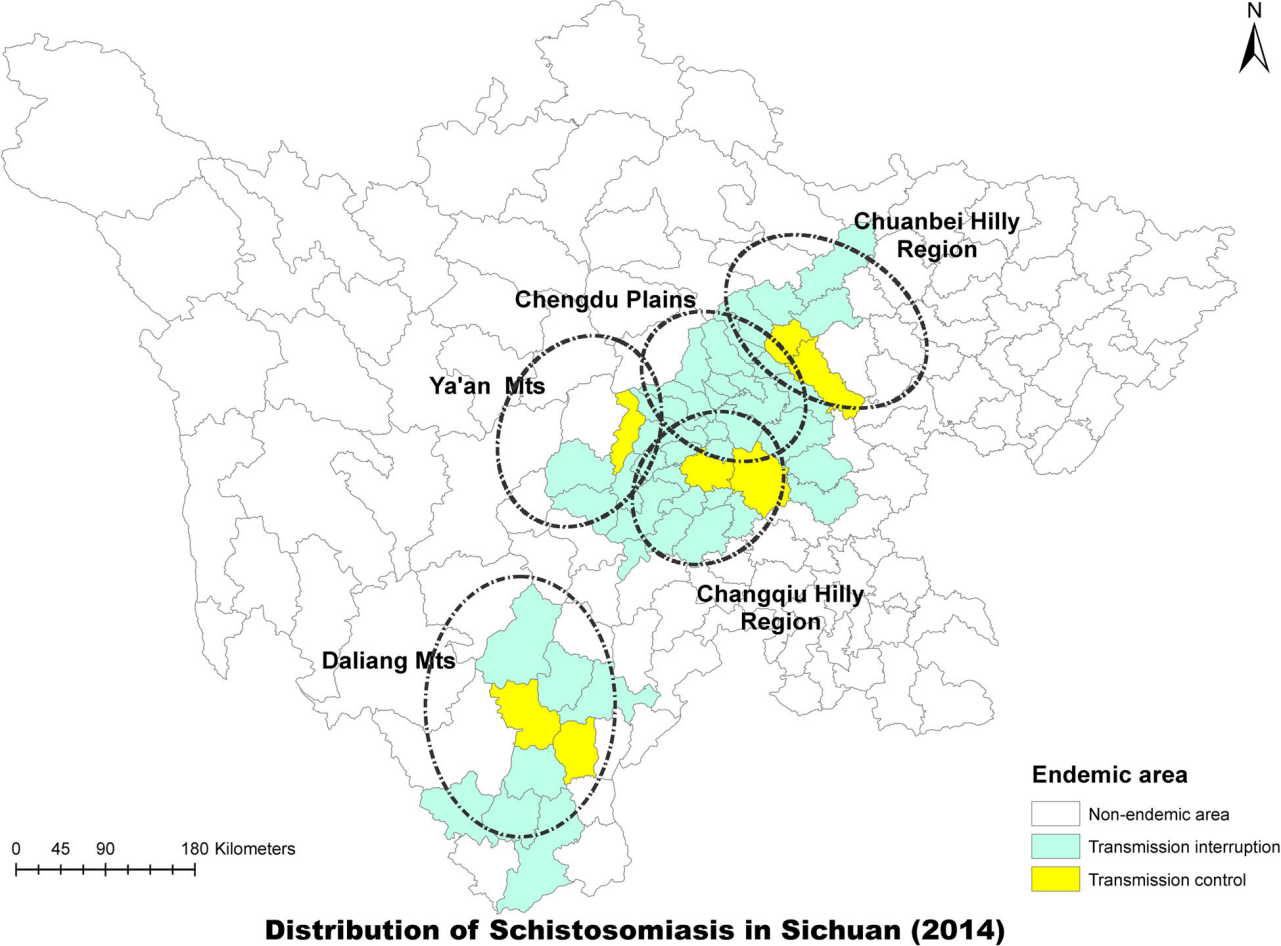
Distribution of schistosomiasis in Sichuan Province in 2014

The 10-year implementation of the integrated strategy from 2005 to 2014 greatly reduced the prevalence of *S. japonicum* in humans, bovines and snails, as well as the acute schistosomiasis cases. By 2014, there were 56 out of the 63 endemic counties achieving the transmission interruption of schistosomiasis and 7 counties achieving transmission control (Table 3; Figs. 4 and 5) [29]. In addition, transmission control of schistosmiasis was achieved in the whole province in 2008.

**Table 3:** Annual number of counties achieving transmission interruption and control in Sichuan from 2005 to 2014

Year	No. counties	No. transmission interruption	No. transmssion control
2005	62	28	25
2006	62	28	30
2007	62	28	34
2008^a^	63	27	36
2009	63	27	36
2010	63	31	32
2011	63	34	29
2012	63	41	22
2013	63	48	15
2014	63	56	7

^a^The total number of endemic counties increased to 63 since 2008, 20 endemic villages were divided to Beichuan Qiang Minority Autonomous County which was not a schistosomiasis endemic area

## Discussion

Over the past six decades, the national schistosomiasis control strategy of China has shifted three times, according to the socio-economic status and epidemiological profiles [30, 31]. The snail control-based integrated control strategy, which was employed from early 1950s to early 1980s, had greatly shrink snail habitats; however, snail cannot be eliminated completely, resulting in frequent re-emergence of schistosomiasis. With the introduction of praziquantel, the national schistosomiasis control strategy of China shifted from transmission control to morbidity control from mid-1980s until 2003. Such a strategy had greatly reduced the prevalence and intensity of *S. japonicum* infection; however, praziquantel cannot prevent re-infection, and humans and livestock may get infections following contact with *S. japonicum*-infested water even if being given chemotherapy with praziquantel. In 2004, a new integrated strategy was developed to control the transmission of *S. japonicum*, through chemotherapy for humans and livestock, snail control, health education, improved sanitation, access to safe water, replacement of bovines with machines, raising bovines in fences [32].

The integrated strategy with emphasis on infectious source control was proposed based on the recognition that bovine is the predominant source of *S. japonicum* infection in the marshland and lake regions [33, 34], and this strategy has been widely proved to be effective for the elimination of schistosomiasis in the marshland and lake regions of China [35–42]. In the survey in 1980s showed that humans were responsible for up to 88% of the schistosome egg excretion to the environment with cattle being responsible for most of the remainder. Dogs and voles have been found to be infected, but contribute little to the transmission cycle due to their low infection rate and small amount of feces in mountainous and hilly regions [43]. However, there is a question about the effectiveness of this strategy for schistosomiasis elimination in the mountainous and hilly regions [44]. Previous studies have demonstrated the short-term effectiveness of the integrated strategy to control the transmission of *S. japonicum* in the mountainous areas of China [45–47]. However, the long-term effectiveness of the new integrated strategy for schistosomiasis elimination remains unknown in the mountainous and hilly regions of China until now.

Since 2005, a 10-year longitudinal study was designed with aims to evaluate the long-term effectiveness of a new government-directed and multi-department integrated strategy on elimination of schistosomiasis in Sichuan Province from 2005 through 2014. To block the transmission cycle of the parasite, supply of safe tap water and the construction of lavatories and latrines were performed, and the coverage of non-hazardous toilets and safe water increased from 58% and 55 to 77 and 97% in the endemic villages, respectively. The 10-year implementation of the integrated strategy resulted in a great reduction in the *S. japonicum* infection in humans, bovines and snails. Since 2007, no acute infection was detected, and no schistosomiasis cases or infected bovines were identified since 2012. In addition, the snail habitats reduced by 62.39% in 2014 as compared to that in 2005, and no *S. japonicum* infection in snails was detected since 2007. By 2014, 88.9% of the endemic counties achieved the transmission interruption of schistosomiasis and transmission control of schistosomiasis was achieved in the whole province in 2008. Our data demonstrate that the integrated strategy is effective for the elimination of schistosomiasis in the mountainous and hilly regions of China.

In 2015, transmission interruption of schistosomiasis was achieved in Sichuan Province [48], and the agenda for schistosomiasis elimination was set by 2023 [49]. There are several challenges for achieving this great goal. (1) Governmental leadership and financial supports. Political commitment and financial support are critically important to the effective schistosomiasis control [22]. Efforts should be made to enhance the leadership and financial supports from various levels of governments. However, the government leadership and financial support may reduce after transmission control and interruption of schistosomiasis is achieved, which may affect the progress towards the elimination of schistosomiasis. (2) The transmission of schistosomiasis is complex, involving social, natural, economic factors. *O. hupensis* snails are usually distributed in complicated mountainous settings, which are difficult for elimination, and bovines are hard to be removed since they have been integrated into local agricultural production and transportation. In addition, numerous wild reservoir hosts complicate the control efforts. (3) Population and bovine migration. There are a large number of humans and livestock migrating from and immigrating into Sichuan Province, which greatly challenges the progress towards the elimination of schistosomiasis. (4) Need of a sensitive and effective surveillance-response system. Currently, schistosomiasis has been reduced to a low-intensity of infection in Sichuan Province, and routine tools are difficult for the identification of human infections. A sensitive and effective surveillance-response system is therefore urgently required for active case finding and rapid response [50–52].

## Conclusions

The results of the present study demonstrate that the government-directed and multi-department integrated strategy is effective for the interruption of schistosomiasis transmission in the mountainous and hilly regions of China.

## Abbreviations

WBLP: World Bank Loan Project for Chinese Schistosomiasis Control Program
IEC: Information, education and communication
